# Long-Service-Life Rigid Polyurethane Foam Fillings for Spent Fuel Transportation Casks

**DOI:** 10.3390/polym16020229

**Published:** 2024-01-14

**Authors:** Zhenyu Zhang, Guangyao Shen, Rongbo Li, Lei Yuan, Hongfu Feng, Xiuming Chen, Feng Qiu, Guangyin Yuan, Xiaodong Zhuang

**Affiliations:** 1 National Engineering Research Center of Light Alloy Net Forming & State Key Laboratory of Metal Matrix Composite, Shanghai Jiao Tong University, 800 Dongchuan Road, Shanghai 200240, China; zhangzhenyu@snerdi.com.cn; 2 Shanghai Nuclear Engineering Research and Design Institute Co., Ltd., 169 Tianlin Road, Xuhui District, Shanghai 200030, China; shenguangyao@snerdi.com.cn (G.S.); lirongbo@snerdi.com.cn (R.L.); hongfu_feng@163.com (H.F.); chenxiuming@snerdi.com.cn (X.C.); 3 The Meso-Entropy Matter Lab, State Key Laboratory of Metal Matrix Composites & Shanghai Key Laboratory of Electrical Insulation and Thermal Ageing, Frontiers Science Center for Transformative Molecules, School of Chemistry and Chemical Engineering, Shanghai Jiao Tong University, 800 Dongchuan Road, Shanghai 200240, China; yuanlei_sherlock@sjtu.edu.cn; 4 School of Chemical and Environmental Engineering, Shanghai Institute of Technology, 100 Haiquan Road, Shanghai 201418, China

**Keywords:** polyurethane foam, fillings, long service life, spent fuel, flame retardance

## Abstract

Soft materials bearing rigid, lightweight, and vibration-dampening properties offer distinct advantages over traditional wooden and metal-based fillings for spent fuel transport casks, due to their low density, tunable structure, excellent mechanical properties, and ease of processing. In this study, a novel type of rigid polyurethane foam is prepared using a conventional polycondensation reaction between isocyanate and hydroxy groups. Moreover, the density and size of the pores in these foams are precisely controlled through simultaneous gas generation. The as-prepared polyurethane exhibits high thermal stability exceeding 185 °C. Lifetime predictions based on thermal testing indicate that these polyurethane foams could last up to over 60 years, which is double the lifetime of conventional materials of about 30 years. Due to their occlusive structure, the mechanical properties of these polymeric materials meet the design standards for spent fuel transport casks, with maximum compression and tensile stresses of 6.89 and 1.37 MPa, respectively, at a testing temperature of −40 °C. In addition, these polymers exhibit effective flame retardancy; combustion ceased within 2 s after removal of the ignition source. All in all, this study provides a simple strategy for preparing rigid polymeric foams, presenting them as promising prospects for application in spent fuel transport casks.

## 1. Introduction

Amid growing concerns regarding global environmental protection, countries are seeking alternative energy sources that do not emit greenhouse gases to replace fossil fuels. In the meantime, the Nino phenomenon exacerbates electricity shortages [1,2]. Currently, renewable energy sources can be divided into solar energy, wind energy, geothermal energy, nuclear power, etc. Among them, nuclear power has much received attention for its high energy density and consistent electricity output [3]. As of now, more than 400 nuclear power reactors are either under operation or under construction worldwide, with the global installed capacity expected to reach 394 gigawatts (GW) [4]. An aspect of the use of nuclear energy is the management of spent fuel, which emits strong hazardous neutron- and gamma-ray radiation. The proper and safe disposal of spent fuel is an unavoidable challenge for the sustained usage of nuclear power [5]. Several countries, including France, China, and Russia, choose the “post-treatment” strategy. In this approach, spent fuel is stored in specialized facilities until its radioactivity reduces sufficiently for reprocessing [6]. The safe transportation of this spent fuel requires specialized casks designed to shield against radioactivity which incorporate multiple layers of materials such as steels, lead, concretes, and polymers [7,8,9]. However, the loading capacity of these specialized casks is much lower than that of traditional containers. Thus, the lightweight design and construction of transportation casks for spent fuel storage remain great challenges.

The spent fuel cask is mainly composed of an outer shell, an anti-vibration layer, a fuel assembly featuring a neutron absorber, and a moderator plate [10]. Among these, the anti-vibration layer occupies a substantial volume in the cask and serves multiple purposes: vibration dampening, buffering, heat insulation, and fire resistance [11]. Generally, timber and honeycomb aluminum are used as anti-vibration materials. However, the energy absorption characteristics of these materials are greatly affected by orientation owing to their intrinsic anisotropy properties [12,13]. Polymeric foams are lightweight materials with the advantages of low cost, tunable structures, ease of processing, and multifunctionality. These materials are commonly used in automotive engineering, thermal insulation, acoustic dampening, biomedical applications, energy storage solutions, and sensor technologies [14,15,16,17,18]. Rigid polyurethane foam, a type of thermosetting polymer resin, has shown promise as a shock absorption layer filling material [19]. Moreover, with a rising strain rate, the energy absorption capability of polyurethane foam increases markedly, and the material becomes stiffer, making it a suitable candidate for the construction of spent fuel transportation casks [20]. However, its anti-vibration performance depends on its density, which consequently affects its weight. Thus, optimizing the trade-off between the anti-vibration performance and weight of rigid polyurethane foam is highly desirable. To the best of our knowledge, few studies have reported on the rational preparation of rigid polyurethane foam as an anti-vibration filling material that would ensure the safety of new spent fuel transport casks. Moreover, scant literature exists on predicting the service lifetime of rigid polyurethane foam through a reliable thermal aging approach.

In this study, rigid polyurethane foams (RPUFs) with different densities and pore sizes were synthesized using a conventional foaming technique. This process involved a reaction between isocyanate and hydroxy groups. The elemental compositions and structures of these rigid polyurethane foams were well characterized by elemental analysis (EA) and Fourier-transform infrared (FTIR) spectroscopy. The morphological and mechanical performance of the foams can be effectively modulated by adjusting the material density. These foams exhibited low water absorption and effective flame-retardant properties. Thermogravimetric analysis revealed that the service lifetime of rigid polyurethane foam is more than 66 years, which meets the design requirements for properly protecting the fuel assembly. This study provides a new path for the development of rigid polyurethane foams with robust mechanical properties, which are suitable for use in spent fuel transportation casks.

## 2. Materials and Methods

### 2.1. Materials

Dihydroxy polyether glycol (PEG-(OH)_2_, Mn = 600), polymeric methylene diphenyl diisocyanate (polymeric MDI, Mn = 388), dimethyl methanephosphonate (DMMP), polysilane, and triethanolamine (Et_3_N) were purchased from Aladdin Reagent (Shanghai, China) and used without purification.

### 2.2. Preparation Procedure

The rigid polyurethane foams were prepared using the in situ foaming method. A mixture of PEG-(OH)_2_, DMMP, distilled water, Et_3_N, and polysilane with a weight ratio of 100:40:0.6:2:0.8 was stirred in a container at 30 °C. Then, this mixture and polymeric MDI were mixed quickly (for 10 s) with the weight ratio of PEG-(OH)_2_ and polymeric MDI of 100:165. Then, this was added to a mold and left for 18 h to achieve the high-density RPUF (HD-RPUF) material.

The procedure and polymerization condition for preparation of other two RPUFs is the same as HD-RPUF. For preparation of middle-density RPUF (MD-RPUF), the weight ratio of PEG-(OH)_2_, polymeric DMI, dimethyl methanephos-phonate, distilled water, Et_3_N, and polysilane in the mixture was 100:155:40:2.6:2:0.8. For preparation of low-density RPUF (LD-RPUF), the weight ratio of PEG-(OH)_2_, polymeric DMI, dimethyl methanephos-phonate, distilled water, Et_3_N, and polysilane in the mixture was 100:150:40:3.4:10:0.2.

### 2.3. Characterization

Fourier Transform Infrared Spectroscopy (FTIR) spectra were recorded on a Spectrum 100 spectrometer (Perkin Elmer, Waltham, MA, USA) with KBr powder. The elemental composition was tested by an elemental analysis (FlashSmart CHNS, Thermo Fisher, Waltham, MA, USA). 

Thermo gravimetric analysis (TGA) spectra were performed on a thermogravimetric analyzer (Q5000IR, TA, Newcastle county, DE, USA) under N_2_ atmosphere from 25 to 600 °C with 20 °C min^−1^. 

Differential scanning calorimetry (DSC) spectra were received from a DSC 6000 spectrometer (Perkin Elmer, Waltham, MA, USA) under N_2_ atmosphere for two heating/cooling cycles from −50 to 180 °C with 10 °C min^−1^. The glass transition temperature (T_g_) was measured according to the standard of ASTM D3418-2015 [21].

Scanning electron microscope (SEM) images were obtained from Sirion-200 field-emission SEM equipment (Field Electron and Ion Company (FEI Co.), Hillsboro, OR, USA). All of the sample was cut into slices, which were used for testing before being coated with gold by a rotary-pumped sputter.

The compression and tensile tests were carried out on a testing machine (model DL2000, EMIC Corp., Tokyo, Japan) following the standards ASTM D 695-15 [22] and ASTM D412 [23], respectively. For compression tests, samples were shaped into cubes with dimensions of 100 mm × 100 mm × 50 mm, and the speed of testing was 1.3 mm min^−1^. For tensile tests, samples were set in dumbbell-shaped specimens, and the speed of testing was 5 mm min^−1^ until fatigue fracture.

Aging prediction tests were performed by TGA analysis from room temperature to 500 °C with different heating rates of 5, 10, 15, and 20 K min^−1^ under an air atmosphere.

## 3. Results

**Scheme 1:**
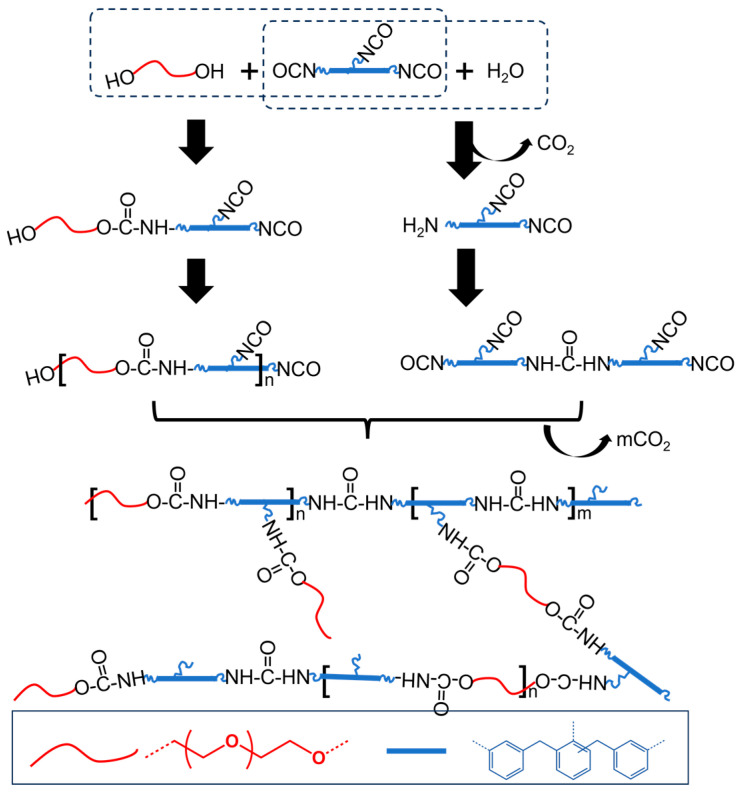
The chemical reactions in as-prepared RPUFs.

As shown in Scheme 1, the rigid polyurethane foams in this study were synthesized using dihydroxy poly(ether polyol) (PEG-(OH)_2_) and polymeric methylenediphenyldiisocyanate (polymeric MDI) as monomers. The synthesis employed a typical polycondensation method and incorporated dimethyl methane phosphonate as a flame retardant, distilled water as a foaming agent, polysilane as a foam stabilizer, and triethanolamine as a catalyst [24]. To improve the thermal aging properties of the polymers, silver nitrate was added to reduce the halogen content. During the polymerization process, CO_2_ blowing from the reaction between water and isocyanate could result in the volume of the material changing rapidly. The volume of the polyurethane foam also expanded in correlation with the increased concentration of the foaming agent, which in turn affected the density of these foams. The densities of the as-prepared polyurethane foams were 0.32, 0.16, and 0.10 g cm^−3^, respectively. Correspondingly, these foams were also designated as HD-RPUF, MD-RPUF, and LD-RPUF. The detailed preparation methods for these polyurethane foams are given in the Section 2.

**Figure 1:**
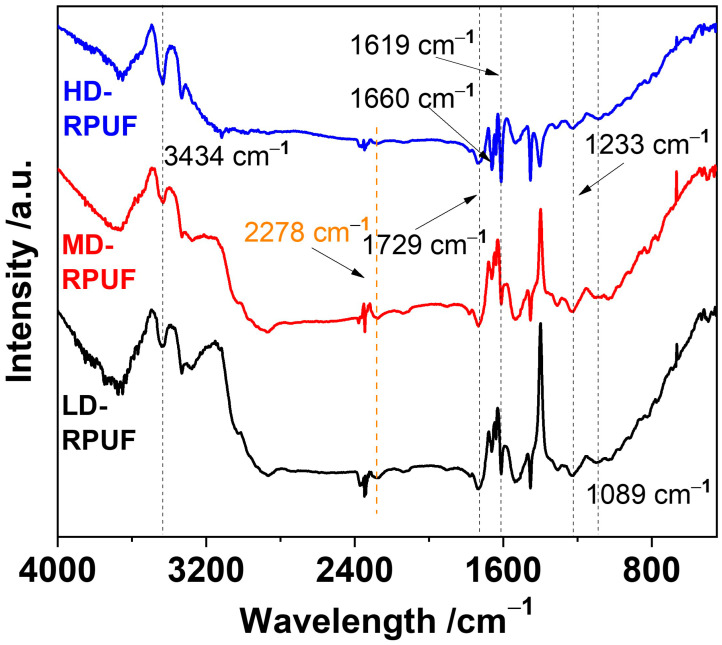
FTIR spectra of HD-RPUF, MD-RPUF, and LD-RPUF.

The structural characteristics of these polyurethane foams were elucidated using FTIR. As depicted in Figure 1, all the samples yielded similar spectral profiles. The peaks located at wavenumbers of 1729, 1619, and 1223 cm^−1^ corresponded to C=O stretching vibrations, C-O stretching in carbonyl groups, and C–N bonds, respectively. These peaks indicate the existence of urethane main linkage in the as-prepared polyurethanes. In addition, the peak at 3434 cm^−1^ corresponded to the N–H bond in urethane [25]. The C=C bonds related to the aromatic groups in the MDI monomer were found at 1660 cm^−1^ [26]. For comparison, the isocyanate (NCO) group was reported at 2278 cm^−1^ [27], which showed very weak peaks in three RPUFs, suggesting that almost all of the polymeric MDI was reacted in the polymerization to form the crosslinking polyurethane foams. Another key monomer of poly(ether polyol) had a strong C-O-C stretching peak at 1089 cm^−1^ [28], which was also found in the as-prepared polyurethane materials. These results indicate the successful synthesis of rigid polyurethane foams using PEG-(OH)_2_ and polymeric MDI. Elemental analysis further validated the structural characteristics with the results listed in Table 1. These results indicate that the three types of polyurethane foams had similar elemental compositions of C, N, and O (Table 2); generally, chlorine, which may generate free radicals that reduce the service life of the material, was present in low amounts. Herein, all of the synthesized materials had low chlorine contents, suggesting their favorable thermal aging performance when used in spent fuel transportation casks.

**Figure 2:**
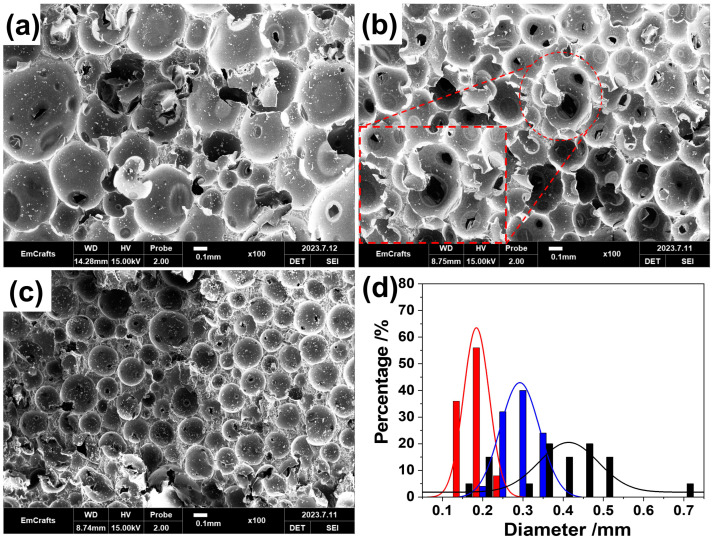
SEM images of LD-RPUF (**a**), MD-RPUF (**b**), and HD-RPUF (**c**); (**d**) size distribution of cells in LD-RPUF (black color), MD-RPUF (blue color), and HD-RPUF (red color), respectively, in micrometer scale.

To evaluate the pore structure of the as-prepared foams, the morphologies of RPUFs with different densities were characterized using a scanning electron microscope (SEM; Figure 2a–c). The SEM images reveal numerous spherical cells with similar obturator structures, indicating they are typical rigid polymeric foams [29]. An enlarged view in Figure 2b clearly displays that these ruptured microspheres have internal hollow spaces, indicating the porous cell structure of the foams. The contents of the foaming agent and foam stabilizer had great influence on the structure of these hollow micro-spheres. With an increasing water content, the generation of CO_2_ blowing increased the volume of the foam size. Moreover, the decrease in foam stabilizer content resulted in the increasing of foam sizes to reach their minimum interfacial tension [30]. Thus, the diameter of the micro-spheres in as-prepared RPUFs increased from 0.185 to 0.415 mm (Figure 2d). Moreover, an increase in the water content led to greater polydispersity in the diameter distribution and introduced discontinuities between the microspheres.

**Figure 3:**
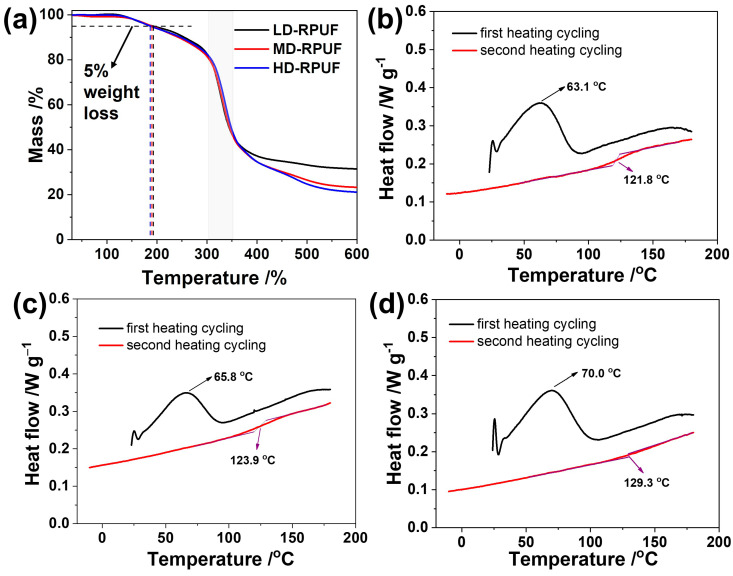
TGA curves of LD-RPUF, MD-RPUF, and HD-RPUF in a nitrogen atmosphere; DSC curves of LD-RPUF (**b**), MD-RPUF (**c**), and HD-RPUF (**d**), respectively, under a nitrogen atmosphere.

The thermal stability of these rigid polyurethane foams was measured at a rate of 10 K min^−1^ in a nitrogen atmosphere. As depicted in Figure 3a, these polyurethane foams exhibited favorable thermal stability up to 400 °C. The temperatures corresponding to a 5% weight loss for LD-RPUF, MD-RPUF, and HD-RPUF were 192.2, 190.3, and 187.0 °C, respectively. Notably, nearly 2% of the thermal weight loss in all three polyurethane foams is attributed to the evaporation of water and other substances with low boiling points from catalysts and side reaction products out of the foams, which occurred at approximately 150 °C [31]. These results indicate that the as-prepared polyurethanes exhibit stable thermal properties at room temperature. The rapid thermal degradation of these foams occurred in the range of 305 to 350 °C, which is attributable to the breakage of the main urethane chains, leading to similar decomposition temperatures [32]. Moreover, 21–31 wt% of residual carbon remained after thermal degradation at temperatures exceeding 600 °C, indicative of their highly crosslinked structure and MDI content.

The differential scanning calorimetry thermographs of the rigid polyurethane foams are presented in Figure 3b–d. In the first heating cycling, the endotherm peaks in the range of 63 °C to 70 °C are associated with the melting of the crystalline structure of RPUFs, which is formed from the ordered soft polyether and hard polymeric MDI segments via hydrogen bonds and π–π interactions [33]. Such broad melting peaks indicate polydispersity in the size and distribution of these crystalline regions. Moreover, the melting point of HD-RPUF is higher than that of MD-RPUF and LD-RPUF, suggesting that the denser structure restricts heat transfer through the polyurethane foam. Due to the foam’s rigid structure, ordered structures form slowly, resulting in the absence of a crystalline peak during the second heating cycling. After eliminating the thermal history of materials in the first heating and cooling cycle, the T_g_ results of our materials were measured carefully. In our system, the T_g_ levels of HD-RPUF, MD-RPUF, and LD-RPUF were over 100 °C, and reached 129.3, 123.9, and 121.8 °C, respectively. The T_g_ result is attributed to the degree of the crosslinking structure of the polymer. Although RPUF with lower density usually has a higher T_g_ point, the content of polymeric MDI in HD-RPUF is slightly higher than that of MD-RPUF and LD-RPUF, which suggests that the degree of crosslinking in HD-RPUF is highest, resulting in the highest T_g_ value.

**Figure 4:**
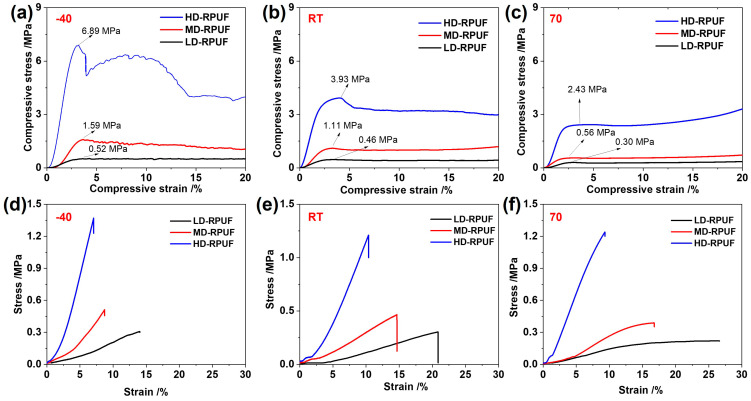
Compression stress–strain curves of LD-RPUF, MD-RPUF, and HD-RPUF under testing temperatures of −40 °C (**a**), 25 °C (**b**), 70 °C (**c**); tensile stress–strain curves of LD-RPUF, MD-RPUF, and HD-RPUF under testing temperatures of −40 °C (**d**), 25 °C (**e**), 70 °C (**f**).

To simulate the operating temperature of rigid polyurethane foams, mechanical tests were conducted at temperatures of −40, 25, and 70 °C. For compression testing, all samples were initially shaped into cubes with width × length × height of 100 × 100 × 50 mm, respectively. Figure 4a–c illustrate that the material volume decreased under applied stress. Using the maximum stress under 10% strain as an indicator of impact resistance, the recorded values for LD-RPUF, MD-RPUF, and HD-RPUF at −40 °C were 6.89, 1.59, and 0.52 MPa, respectively. These values meet the design requirements for compression stress in spent fuel transport tanks, which is attributable to their occlusive structure and rigid polymeric backbone, such as MDI. As expected, HD-RPUF, with the highest density, also exhibited the highest compression resistance. These trends were consistent at test temperatures of 25 and 70 °C. According to classical polymer physics, molecular chain and segment mobility in the material increase with rising temperatures. Moreover, according to the DSC results, the melting of crystalline domains in the polyurethane foam occurred at temperatures above 25 °C, leading to reduced compressibility [34]. For example, as the temperature shifted from −40 to 70 °C, the compression stress in HD-RPUF was reduced to 2.43 MPa, indicating the softening of the polyurethane foams at higher temperatures.

For the tensile test, the representative stress–strain curves of LD-RPUF, MD-RPUF, and HD-RPUF are shown in Figure 4d–f. For example, when tested at −40 °C, the stress–strain curves for these materials initially exhibited a linear increase at low strain, suggesting typical elastic deformation. As the strain continued to increase, the stress increased sharply until fracture, which is attributable to both the deformation of crystallization domains within the alignment of the structural backbone and the crosslinking structure [35]. The tensile stress for HD-RPUF was 1.37 MPa, exceeding that of MD-RPUF (0.51 MPa) and LD-RPUF (0.31 MPa). However, the maximum strain increased with the decreasing density of polyurethane foam. This phenomenon suggests that foams with larger pore sizes exhibit excellent stretchability under stress, leading to greater material strain [36]. When the test temperature increased, HD-RPUF displayed the highest elongation at fracture, whereas LD-RPUF exhibited the highest tensile strain. In addition, tensile values at the point of elongation remain stable at different testing temperatures, indicating that material fracture is highly dependent on the inherent intermolecular interaction of polymer chains. Conversely, elongation strains at the point of fracture extend as test temperatures increase [37], which can be attributed to the enhanced molecular chain and segment mobility of the material. Based on the tensile testing, the Young’s modulus, a mechanical property of polymeric materials, was measured from the ratio of the stress to axial strain within the linear elastic deformation range. The equation is defined as follows:

(1) E=σ/ε,

where *E* is Young’s modulus, *σ* is a force applied to the material divided by the cross-sectional area, and *ε* is the proportional deformation in the material. 

Similar to the tensile strength of the as-prepared RPUFs, the Yong’s modulus of HD-RPUF was higher than that of MD-RPUF and LD-RPUF, due to their having the highest crosslinking structure. Furthermore, the molecular motion of the structure could quickly change when the temperature increased. Thus, the Yong’s modulus values of these three RPUFs decreased when the temperature increased from −40 °C to 70 °C. All of mechanical properties of three as-prepared RPUFs has been listed in the Table 3.

**Figure 5:**
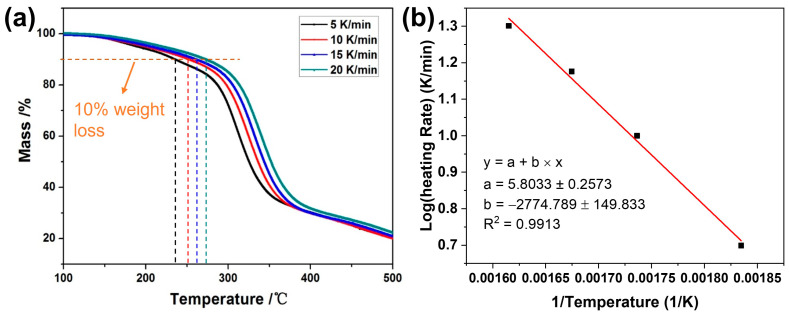
TGA curves of LD-RPUF at different heating rates (**a**); linear fitting between the logarithm of the heating rates and the reciprocal of *T*_10%_ (1/T) from TGA results (**b**).

Owing to the similar thermal stabilities of the as-prepared materials, the lifetime of these polyurethane foams was evaluated using thermal aging tests, with LD-RPUF serving as the model. Figure 5a displays the thermogravimetric analysis (TGA) curves illustrating the thermal decomposition of LD-RPUF from room temperature to 500 °C under the different heating rates, including 5, 10, 15, and 20 K min^−1^. In Figure 5a, these polyurethane foams exhibit stable thermal properties at <150 °C in an air atmosphere. Rapid degradation occurs at temperatures exceeding 300 °C, a threshold lower than that observed in a nitrogen atmosphere; this may be attributable to accelerated oxidation in the existence of oxygen. As the heating rate increases, the rate of thermal degradation of LD-RPUF decreases, which is likely due to the heat diffusion hysteresis phenomenon in the material [37].

Using 10% weight loss in the TGA as the failure criterion [38], the *T*_10%_ temperatures for LD-RPUF in kelvin were 545.03 K, 575.94 K, 597.17 K, and 619.15 K at the heating rates of 5, 10, 15, and 20 K min^−1^, respectively. The relationship between the logarithm of the heating rates (*log β*) and the reciprocal of *T*_10%_ (1/*T*) is given in Figure 5b. The fitted line exhibits a strong linear correlation. A coefficient of determination (R2) of 0.9913 was obtained [39]. The Flynn–Wall–Ozawa equation was employed to calculate the apparent activation energies of polymeric materials in accordance with the ASTM E1641-07 standards [40,41]. The equation is defined as follows:

(2) $$E=-\left(R/b\right)\times \Delta \left(\log\beta \right)/\Delta \left(1/T\right),$$

where *R* is the universal gas constant (8.314 J mol^−1^·K^−1^), *b* is a differential approximation, and Δ(*log β*)/Δ(1/*T*) is the slope of the logarithm of the heating rates and the reciprocal of *T*_10%_. The activation energy (*E*) of 104.89 kJ mol^−1^ was obtained with the calculation of Equation (2).

Thus, the predicted lifetime of LD-RPUF can be obtained using the ASTM E1877 standards [42] and is given by the following equation:

(3) $$\log t_{f}=E/\left(2.303RT_{f}\right)+\log\left[E/\left(R\beta \right)\right]-a$$

where *t_f_* is the service lifetime, *T_f_* is the service temperature, and *a* is an integral approximation.

At the maximum service temperature of 30 °C, the predicted lifetime of the LD-RPUF foam is 95 years. Thus, the actual service lifetime of these polyurethane foams would be 66 years according to the safety coefficient of 0.7 [43], which exceeds the standard service lifetime of 30 years for spent fuel transport casks. 

**Figure 6:**
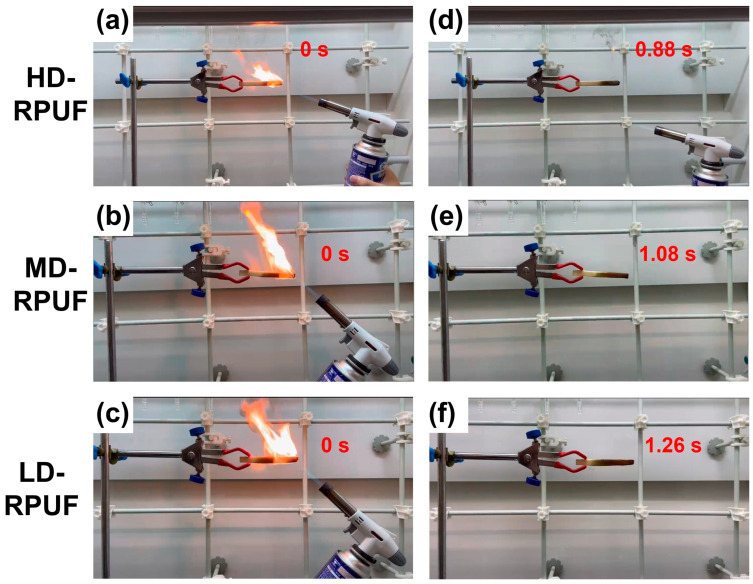
Flame-retardant performances of LD-RPUF, MD-RPUF, and HD-RPUF before (**a**–**c**) and after (**d**–**f**) removing of ignition source.

Flame retardancy tests were conducted for the as-prepared polyurethane foams, with the results summarized in Table 2 and Figure 6. After ignition, flames spread rapidly across the material surfaces, accompanied by black smoke. Digital photos taken during the tests reveal no evidence of melt dripping from the as-prepared polyurethane foams. Once the ignition source was removed, the combustion of the as-prepared materials ceased quickly before complete carbonization could occur. Figure 6d–f indicate that the combustion of these polyurethane foams ceased within 2 s, suggesting that phosphonate compounds effectively prevent re-ignition during the flame tests [44]. Moreover, the carbon residue ratios exceeded 90%, further demonstrating the high flame-retardant efficiency of these rigid polyurethane foams.

## 4. Conclusions

This study introduced a novel class of rigid polyurethane foams formulated with phosphonate compounds as the flame retardant. These foams were synthesized through a standard polycondensation reaction involving dihydroxy PEG and MDI. The structures and morphologies of these polyurethane foams were characterized using FTIR and SEM. By modulating the water content, the density and size of cells could be adjusted. These foams exhibited robust mechanical properties, characterized by a compression stress of 6.89 MPa and a tensile stress of 1.37 MPa; unique thermal stability, indicated by *T*_5%_ > 185 °C; and excellent flame retardancy, marked by a flame extinction time of <2 s. More importantly, the predicted service lifetime for these polyurethane foams is 66 years, exceeding the standard service lifetime of 30 years for conventional spent fuel transportation casks. This study provides a simple method for creating rigid, lightweight, and vibration-resistant soft materials suitable for the manufacture of spent fuel transport casks.

## Figures and Tables

**Table 1:** Reaction composition of as-prepared polyurethane foams.

Sample	PEG-(OH)_2_ (kg)	Polymeric MDI (kg)	DMMP (kg)	Distilled Water (kg)	Et_3_N (kg)	Polysilane (kg)	Molar Ratio of NCO in Polymeric DMI to OH in PEG-(OH)_2_
HD-RPUF	100	165	40	0.6	2	0.8	3.70
MD-RPUF	100	155	40	2.6	2	0.8	3.47
LD-RPUF	100	150	40	3.4	10	0.2	3.36

**Table 2:** Element content and flame retardancy properties of as-prepared polyurethane foams.

Sample	Contents of Elements (wt%) ^(a)^								Flame Extinction Time (s)	Residual Carbons (wt%) ^(b)^
	C	H	N	O	P	Si	Cl	Other		
HD-RPUF	61.82	6.04	7.13	14.7	1.125	0.078	0.005	<1	0.88	96.71 ± 1.70%
MD-RPUF	62.73	6.13	7.27	13.6	0.905	0.088	0.005	<1	1.08	92.48 ± 0.77%
LD-RPUF	62.51	6.07	7.36	13.2	1.736	0.021	0.004	<1	1.26	90.64 ± 1.44%

^(a)^ elemental composition tested by elemental analysis measurement; ^(b)^ ratio of weight after flame tests.

**Table 3:** Mechanical property data of LD-RPUF, MD-RPUF, and HD-RPUF under different testing temperatures.

Sample	Compression Strength (MPa)			Tensile Strength (MPa)			Strain (%)			Yong’s Modulus (MPa)		
	−40	25	70	−40	25	70	−40	25	70	−40	25	70
HD-RPUF	6.89	3.93	2.43	1.37	1.24	1.21	7.06	9.25	10.32	19.41	13.48	11.72
MD-RPUF	1.59	1.11	0.56	0.51	0.47	0.39	8.70	14.58	16.74	5.86	3.22	2.33
LD-RPUF	0.52	0.46	0.30	0.31	0.31	0.22	14.01	20.83	26.57	2.21	1.49	0.83

## Data Availability

The data presented in this study are available on request from the corresponding author.
